# Supplementary material for: The Construction and Exploration of a Comprehensive MicroRNA Centered Regulatory Network in Foxtail Millet (Setaria italica L.)
Source: Front Plant Sci. 2022 May 6;13:848474. doi: 10.3389/fpls.2022.848474 (PMC9121102; doi:10.3389/fpls.2022.848474)
Supplement: Supplementary file 4 [file Data_Sheet_4.zip › Sit-miRNovel4_Seita.6G095500.1_323_TPlot.pdf]

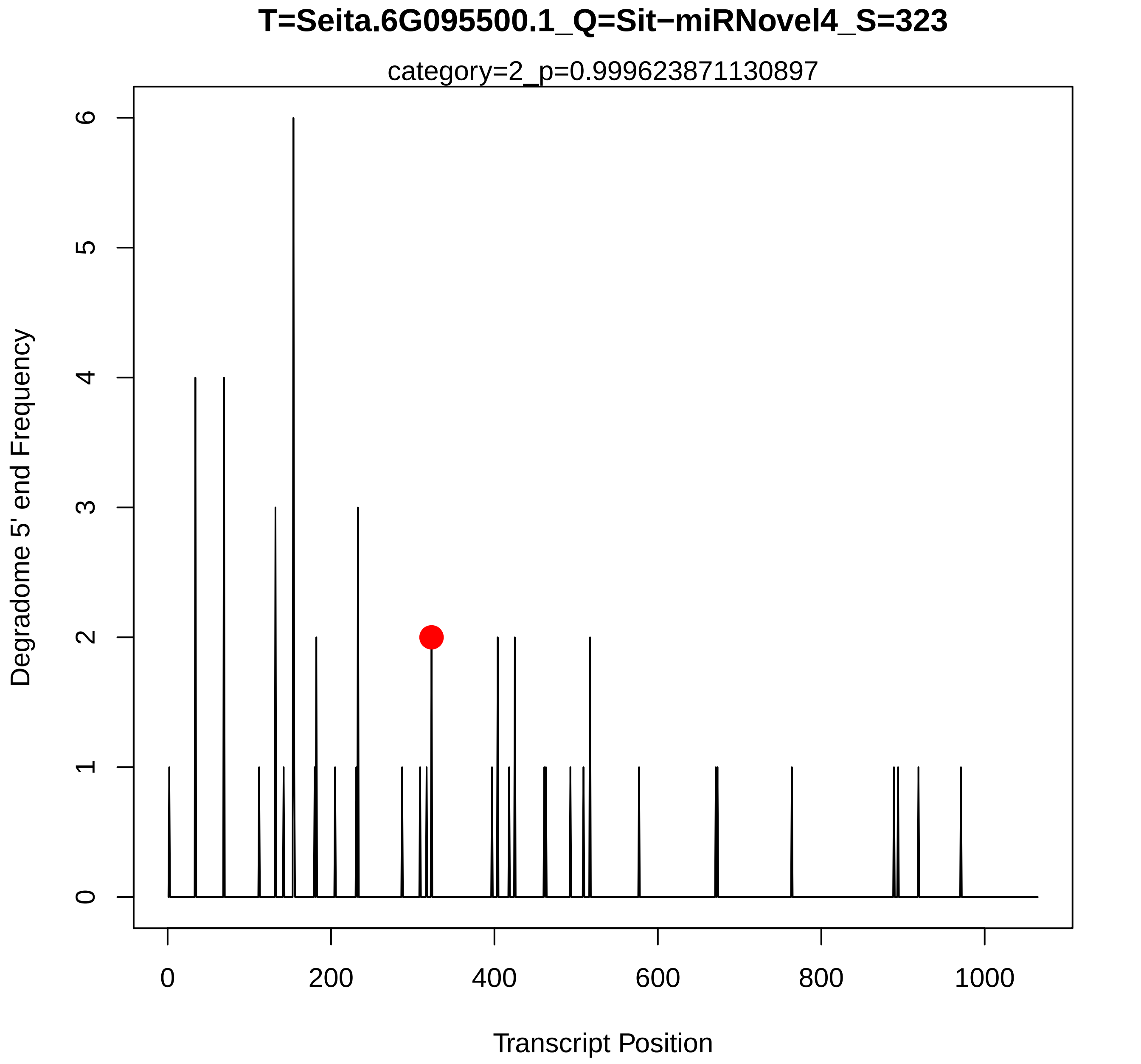

T=Seita.6G095500.1_Q=Sit−miRNovel4_S=323
category=2_p=0.999623871130897
Degradome 5' end Frequency
0
1
2
3
4
5
6
0
200
400
600
800
1000
Transcript Position
